# Aberrantly reduced expression of miR-342-5p contributes to CCND1-associated chronic myeloid leukemia progression and imatinib resistance

**DOI:** 10.1038/s41419-021-04209-2

**Published:** 2021-10-05

**Authors:** Yi-Ying Wu, Hsing-Fan Lai, Tzu-Chuan Huang, Yu-Guang Chen, Ren-Hua Ye, Ping-Ying Chang, Shiue-Wei Lai, Yeu-Chin Chen, Cho-Hao Lee, Wei-Nung Liu, Ming-Shen Dai, Jia-Hong Chen, Ching-Liang Ho, Yi-Lin Chiu

**Affiliations:** 1 Division of Hematology and Oncology Medicine, Department of Internal Medicine, Tri-Service General Hospital, National Defense Medical Center, 11490 Taipei, Taiwan, ROC; 2 Department of Biochemistry, National Defense Medical Center, 11490 Taipei, Taiwan, ROC; 3 Graduate Institute of Life Sciences, National Defense Medical Center, 11490 Taipei, Taiwan, ROC

**Keywords:** Chronic myeloid leukaemia, Oncogenes

## Abstract

Chronic myeloid leukemia (CML) is a myeloproliferative disorder associated with the Philadelphia chromosome, and the current standard of care is the use of tyrosine kinase inhibitors (TKI). However, some patients will not achieve a molecular response and may progress to blast crisis, and the underlying mechanisms remain to be clarified. In this study, next-generation sequencing was used to explore endogenous miRNAs in CML patients versus healthy volunteers, and miR-342-5p was identified as the primary target. We found that miR-342-5p was downregulated in CML patients and had a significant inhibitory effect on cell proliferation in CML. Through a luciferase reporter system, miR-342-5p was reported to target the 3’-UTR domain of CCND1 and downregulated its expression. Furthermore, overexpression of miR-342-5p enhanced imatinib-induced DNA double-strand breaks and apoptosis. Finally, by analyzing clinical databases, we further confirmed that miR-342-5p was associated with predicted molecular responses in CML patients. In conclusion, we found that both in vivo and in vitro experiments and database cohorts showed that miR-342-5p plays a key role in CML patients, indicating that miR-342-5p may be a potential target for future CML treatment or prognostic evaluation.

## Introduction

Chronic myeloid leukemia (CML) is a clonal myeloproliferative disease with an incidence of 1–2 cases per 100,000 adults [1]. Current research suggests that the predominant cause of most CML occurrences is a long-arm translocation between chromosomes 9 and 22, also known as the Philadelphia chromosome [2]. Primarily, BCR-ABL fusion proteins act as active tyrosine kinases that promote cell growth or prevent apoptosis by perturbing downstream pathways [3]. Given the enormous impact of BCR-ABL in causing CML, the treatment of this disease has shifted from conventional therapy to targeting this fused tyrosine kinase, such as the significant clinical breakthroughs achieved with imatinib [4]. However, not every patient achieves an optimal response after tyrosine kinase inhibitor (TKI) treatment.

Mature microRNAs consist of short non-coding RNA molecules (20-22 nucleotides) that can affect the stability of target gene mRNAs by binding to miRNA binding sites in plants and animals [5]. MicroRNAs are involved in hematopoiesis through complete genetic modifications. For example, specific miRNAs regulate mandatory genes in hematopoiesis [6]. Furthermore, each hematopoietic lineage may be held by miRNA clusters, such that erythropoiesis is promoted by miR-16, miR-144, and miR-451 and downregulated by miR-150, miR-155, miR-221, and miR-222 [7]. It has even been reported that hematopoietic stem cells (HSCs) are maintained by miR-126 and miR-142, while abnormal expression of miR-29a can promote the proliferation of HSCs [8]. In addition, the expression of some microRNAs is associated with leukemogenesis and they behave like oncogenes or tumor suppressors, such as miR-125a/b and miR-193a [9]. Specific microRNAs may function to regulate BCR-ABL expression in CML patients and influence resistance to tyrosine kinase inhibitors, further affecting the prognosis or pathogenesis of CML patients [10].

To investigate the potential association between CML and unidentified miRNAs, we examined peripheral blood mononuclear cells (PBMC) from CML patients and healthy donors. Several miRNAs were found to be significantly differentially expressed, in particular, miR-342-5p was significantly downregulated in CML patients. MiR-342-5p is an intrinsic microRNA found in the host gene Ena-vasodilation-stimulating phosphoprotein (EVL) and has been reported as a possible tumor suppressor gene [11]. Overexpression of miR-342-5p in leukemia cells significantly suppressed BCR-ABL expression and cell viability. Further analysis revealed that Cyclin D1 (CCND1) was one of the target genes directly repressed by miR-342-5p. Expression of miR-342-5p may inhibit CCND1 expression and reduce the proliferation and DNA repair of leukemic cells, further sensitizing them to imatinib. This phenomenon was further validated in an in vivo model. Further association of miR-342-5p upregulated gene-set with disease progression was observed in the clinical CML database. In conclusion, our findings support the potential role of miR-342-5p in predicting response to TKI therapy.

## Methods

### Cultures of CML cell lines

Human CML cell lines K562, MEG01, and KU812 were obtained from the Bioresource Collection and Research Center (BCRC, Hsin-chu, Taiwan). Cells were cultured in RPMI-1640 medium supplemented with 10% heat-inactivated fetal bovine serum (2 mM glutamine, 1% penicillin, and streptomycin), and cultured at 37 °C in a humidified incubator with 5% CO_2_.

### Total and MicroRNA extraction and quantification

Total RNA was isolated from CML cell lines and lysis by TRIzol reagent (Thermo-Fisher Scientific Inc., Waltham, MA) according to the protocol of the manufacturer. MicroRNA extraction was performed according to the miRNeasy Mini Kit (Qiagen, Hilden, Germany). Complementary DNA was synthesized from 1 µg of total isolated RNA using the miScript II RT Kit (Qiagen). Real-time PCR was performed using the SYBR Green system with the ABI 48-well Step-One TM Real-Time System (Applied Biosystems, Foster City, CA), and CT values of each sample were determined. U6 spliceosomal RNA was used for miRNA normalization.

### Preparation of peripheral blood samples from CML patients and small RNA sequencing

This study was approved by the Institutional Review Board of Tri-Service General Hospital (1-105-05-052). A total of 20 CML patients and 13 healthy controls were included, and peripheral blood samples (6–8 ml) were drawn after written informed consent was obtained. The clinical parameters related to CML patients are shown in supplemental table 1. The monocyte fraction was isolated by Ficoll-paque plus (Sigma-Aldrich, Munich, Germany), and total RNA was extracted. Next-generation sequencing library preparations were constructed according to the manufacturer’s protocol. The sequences were processed and analyzed by GENEWIZ (South Plainfield, NJ, USA).

### MTAM-based CML xenograft animal model

The MTAM system was prepared as previously described [12, 13]. All animal experiments are conducted following the guidelines of the Laboratory Animal Center in National Defense Medical Center. MTAM was implanted in the back of a 7-week-old C57BL/6JNarl mouse (five per experimental group), and the wound was sealed with surgical sutures. Mice implanted with MTAM were randomly divided into two groups, and oral administration of imatinib (100 mg/kg, BID) or vehicle of the same volume was started two days after implantation. On the 15th day, mice were sacrificed, and the subcutaneously implanted MTAM was accessed by cutting skin from the abdomen to the back. The formations of blood vessels on the MTAM were photographed. The MTAM was then partitioned into three aliquots, cut up and soaked in 0.5% MTT (Sigma-Aldrich) solution for 2–4 h. After dissolving the crystals by DMSO (Sigma-Aldrich), samples were analyzed by an ELISA reader (SpectraMax iD3, Molecular Devices, CA).

### Western blot analysis and phosphorylation array

Sample preparation as previously described [14]. The primary antibodies at the indicated dilutions: c-Abl (K12; Santa-Cruz Cat#sc-131), β-actin (Sigma-Aldrich Cat#A5441), phospho-ATR-S428 (ABclonal Inc., USA; Cat#AP0676), phospho-chk1-S345 (ABclonal Cat#AP0578), phospho-ATM-S1981 (ABclonal Cat#AP0008), phospho-chk2-Thr68 (Cell Signaling Technology [CST] Cat#2197), GAPDH (CST Cat#5174), CCND1 (A12; Santa-Cruz Cat#sc-8396), CCNE (C19; Santa-Cruz Cat#sc-198), E2F1 (C20; Santa-Cruz Cat#sc-193), p27[Kip1] (BD Cat#610242), RB (IF8; Santa-Cruz Cat#sc-102), PARP (CST Cat#9542), Bcl-xL(H-5) (H5; Santa-Cruz Cat#SC-8392). About the phosphorylation array, the Proteome Profiler Human Phospho-MAPK Array Kit (ARY002B; R&D Systems, Minneapolis, MN) was used according to the manufacturer’s instructions.

### Transfection for miRNA, CCND1, and miRNA inhibitor

For transfection, K562 cells were seeded in 6 well culture plates in RPMI medium containing 10% FBS. Mimics miR-342-5p, miR-NEG control (up to 20 nM), miR-342-5p inhibitor (50 nM) as well as CCND1/pCDNA3.1(+) plasmid (3 μg) were transfected using the lipofectamine 3000 reagent kit (L3000015, Thermo-Fisher Scientific) according to the manufacturer’s instructions.

### Cell viability assay, soft agar colony assay, and NC-3000 for cell proliferation and cell cycle

Cell viability assay was studied through XTT assay (Sigma-Aldrich) and trypan blue dye (Sigma-Aldrich) exclusion assay, where the cells that took up trypan blue were counted as dead. Cells stained with trypan blue and evaluated under a microscope using a hemocytometer. Soft agar colony formation was performed according to the previously described with minor modifications [15]. NucleoCounter^®^ NC-3000 (Chemometec, Denmark) was utilized for cell cycle analysis following the manufacturer’s instructions.

### Assessment of apoptosis by Annexin-V/PI staining

Cells were stained with annexin-V-FITC/PI kit following the manufacturer’s instruction (BD). Stained cells were analyzed on a FACScalibur with CellQuest software (BD).

### Luciferase assay for detect miRNA-binding sites in the 3′-UTR

For luciferase reporter assays, K562 cells were transfected in 96-well plates with 20 nM miRNA duplex or miRNA hairpin precursors (Thermo-Fisher Scientific), 0.4 µg 3′UTR-luciferase vector (Origene, Rockville, MD), and 0.01 µg Renilla vector (pRL-TK) using Lipofectamine 3000. Forty-eight hours after transfection, luciferase activity was measured using the Dual-Glo Luciferase Assay (Promega, WI).

### Identification of the impacted DEGs by miR-342-5p expression

Microarray analysis was performed using Phalanx Human OneArray^®^ service (data available in supplemental table 2 or accessing GEO accession number: GSE171659). The ClueGO app in Cytoscape was applied to build a differentially expressed gene ontology biological process (GOBP) terminology network [16, 17]. The difference between miR-NEG or miR-342-5p expression with and without imatinib treatment was further analyzed by GSEA with default settings [18]. The gene sets with separated GOBP enrichment trends were visualized by Enrichment map and clustered and auto-annotated by the degree of overlapping among gene sets [19].

### Gene expression profiling and GSVA scoring

Gene expression profile data were publicly accessible from the NCBI website with accession numbers GSE4170 (119 patients), GSE13204 (96 patients), GSE130404 (76 CML patients), and GSE144119 (97 samples) [20–24]. In addition, Single-cell gene expression matrix data of GSE76312 were directly downloaded from the NCBI GEO website [25]. The clinical staging or phenotype classification of CML patients was obtained from the authors’ data. The GSVA (Gene Set Variance Analysis) package in R was used to score individual CML clinical samples with default settings [26].

## Results

### A comprehensive evaluation of miRNAs expressed aberrantly in the blood samples of CML patients

**Fig. 1:**
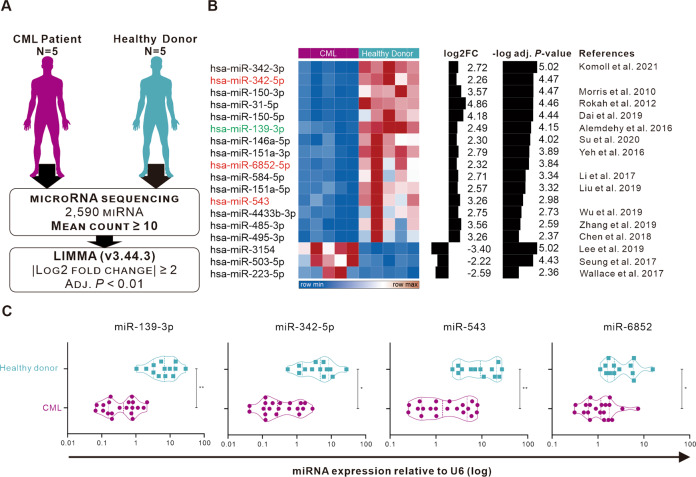
Comprehensive analysis of miRNAs with abnormal expression in CML patients using miRNA sequencing. **A** Schematic diagram of the screening process for miRNAs with significant differential expression using microRNA sequencing data; (**B**) Heat map of miRNA expression of 18 miRNAs with significant differences of 4-fold or more. The red text indicates that the candidate miRNA has not been discussed for its potential mechanism or role in leukemia. Green text means that the miRNA has been confirmed in several papers for its role in leukemia. **C** The expression of candidate miRNAs in PBMC of CML patients and healthy donors was confirmed by qPCR, and U6 was used as an internal control. Each data represents mean ± SD from independent experiments performed in triplicate. Statistical significance analysis was performed with Student’s *t*-test. *P < 0.05; **P < 0.01.

To evaluate the aberrant expression of miRNAs in blood samples from CML patients, we initially collected RNA samples from five chronic phase CML patients and healthy donors, respectively, for miRNA sequencing. Further LIMMA analysis of the differentially expressed miRNAs narrowed the results to 18 miRNAs, with 15 significantly reduced and three significantly increased in CML patients. (Fig. 1A). Most of them have been reported to be associated with tumorigenesis, such as miR-342-3p, miR-150-5p, miR-151-3p, miR-151-5p, miR-584-5p, miR-485-3p, and miR-495-3p have been evaluated for their potential targets and mechanisms in solid tumors [27–34]. The miR-139-3p, miR-31-5p, miR-150-3p, miR-146a-5p, miR-4433-3p, miR-3154, miR-503-5p and miR-223-5p have been studied for their tumor-suppressive role in hematological cancers [35–42]. The miR-342-5p, miR-6852-5p, and miR-543 have not been confirmed to be associated with hematological cancers (Fig. 1B). Further analysis by qPCR of blood samples from 20 CML patients (including the 5 cases mentioned above) and 13 healthy donors showed that the expression of these four miRNAs (miR-139-3p as positive control) was significantly reduced in CML patients (Fig. 1C).

### MiR-342-5p shows the most potent inhibitory effect on CML proliferation

**Fig. 2:**
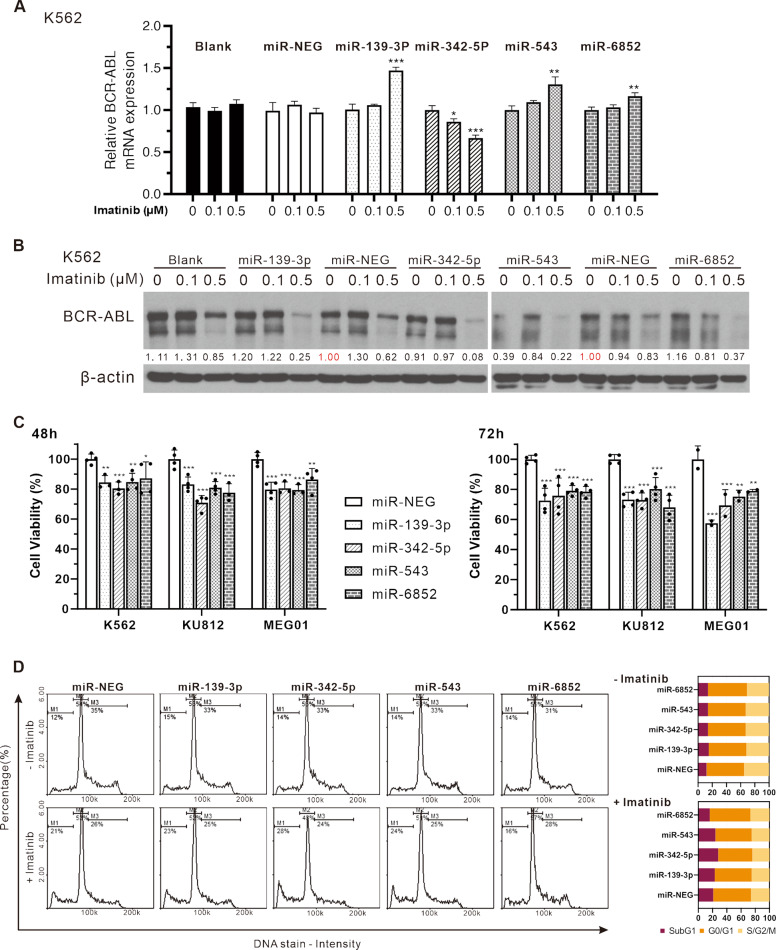
Assessment of the effect of four miRNAs on cell viability and BCR-ABL expression in the presence or absence of imatinib treatment in various CML cell lines. Effect of imatinib on BCR-ABL expression by qPCR (**A**) and western blot (**B**) in the expression of four miRNAs. **C** The effects of adding four miRNAs at 48 and 72 hours on CML proliferation were presented by XTT assay. A one-way ANOVA approach was used to assess the significance of the effects of each miRNA compared with miR-NEG. Each data represents mean ± SD from independent experiments performed in triplicate. *P < 0.05; **P < 0.01; ***P < 0.001. **D** The effect of miR-NEG and four miRNAs on cell cycle distribution of K562 was evaluated by NC-3000^TM^ NucleoCounter^®^ (ChemoMetec, Denmark) with or without imatinib (0.5 μM, 48 h) treatment, and the results quantified for different phases are shown on the right.

Aberrantly elevated expression of BCR-ABL is thought to have the most significant impact on CML proliferation and imatinib resistance [43]. We, therefore, evaluated the effects of expressing miR-139-3p, miR-342-5p, miR-543, and miR-6852 on BCR-ABL expression in the K562 CML cell line. Results showed that when cells expressed miR-342-5p, mRNA and protein expression of BCR-ABL decreased with increasing doses of imatinib treatment (Fig. 2A, B). In the cell viability assay, although all of these four miRNAs significantly inhibited the viability of multiple CML cell lines (K562, KU812, and MEG01), miR-342-5p showed comparable inhibition to miR139-3p (Fig. 2C). Furthermore, it increased subG1 the most in cells subjected to 0.5 µM Imatinib (Fig. 2D), suggesting that miR-342-5p can affect CML cell survival and tolerance to imatinib by inhibiting the expression of BCR-ABL.

### MiR-342-5p targeted the 3′UTR domain of CCND1 mRNA to downregulate its expression

**Fig. 3:**
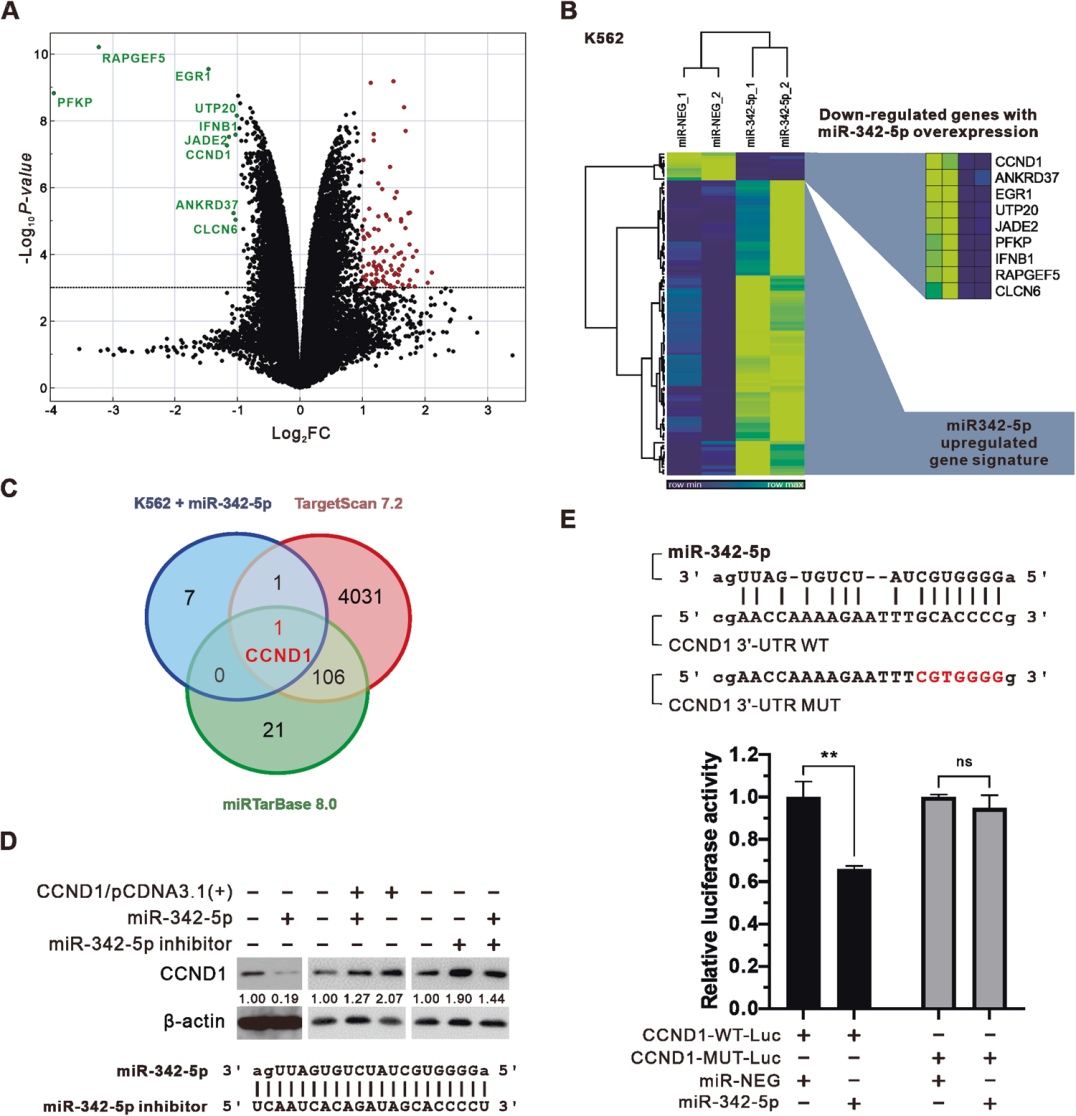
Combining experimental validation and prediction of miRNA target database with microarray analysis to find CCND1 as the target of miR-342-5p. **A** Volcano plot showing the distribution of differentially expressed genes with miR-342-5p expression. The red dots represent significantly upregulated genes, and the green represents significantly downregulated genes. Filtering criteria: |Log_2_FC | ≧ 1, −Log_10_ *p*-value >3. **B** Heat map presents the relative expression and hierarchical clustering of differentially expressed genes with or without miR-342-5p expression. **C** Venn diagram presents the results of the intersection of K562 expressing miR-342-5p downregulated gene (blue circle), TargetScan predicting miR-342-5p target gene (red circle), and miRTarBase experiment validated miR-342-5p target gene (green circle). **D** Western blot to confirm the expression of CCND1 affected by miR-342-5p (20 nM) or miR-NEG (20 nM) transfection combined with CCND1/pCDNA3.1(+)(3 μg) or miR-342-5p inhibitor (50 nM). The sequence of the utilized miR-342-5p inhibitor is shown below. **E** The miRANDA-predicted miR-342-5P sequence is aligned with the CCND1 3′-UTR WT target sequence. Sequences with manipulated mutations in the CCND1 3′-UTR MUT are shown in red. Luciferase activity assay shows the relative intensity of luminescence generated by CCND1-WT-Luc or CCND1-MUT-Luc (0.4 μg) under miR-342-5p or miR-NEG expression (20 nM). Student’s *t*-test assessed the statistical significance of the difference between miR-342-5p and miR-NEG, ns: no significant differences; **P < 0.01.

To resolve the effect of miR-342-5p in CML, we performed microarray analysis and distinguished differentially expressed genes into down- and upregulated groups. Genes significantly downregulated under miR-342-5p expression were CCND1, ANKRD37, EGR1, UTP20, JADE2, PFKP, IFNB1, RAPGEF5, and CLCN6 (Fig. 3A). On the other hand, more genes may be upregulated by the indirect effects of miR-342-5p, defined as “miR-342-5p upregulated gene signature” (Fig. 3B). For the nine downregulated genes, further cross-comparison of two miRNA target databases, TargetScan 7.2 and miRTarBase 8.0, with Venn diagrams to find potential targets for miR-342-5p revealed that only CCND1 was a common target for all (Fig. 3C) [44, 45]. Furthermore, its inhibitory effect on CCND1 was further bi-directionally verified by western blot (Fig. 3D). Moreover, we used miRANDA to predict the potential targets of miR-342-5p on CCND1 mRNA sequences and found that the specific sequence at the 3’UTR of CCND1 mRNA had the highest complementarity with miR-342-5p (Fig. 3E) [46]. The results showed that only the relative luciferase activity of CCND1-WT-Luc was decreased after the addition of miR342-5p rather than CCND1-MUT-Luc (Fig. 3E), demonstrating the inhibitory role of miR-342-5p on CCND1 by targeting this sequence.

### Genes upregulated by miR-342-5p were associated with negative regulation of leukocyte proliferation

**Fig. 4:**
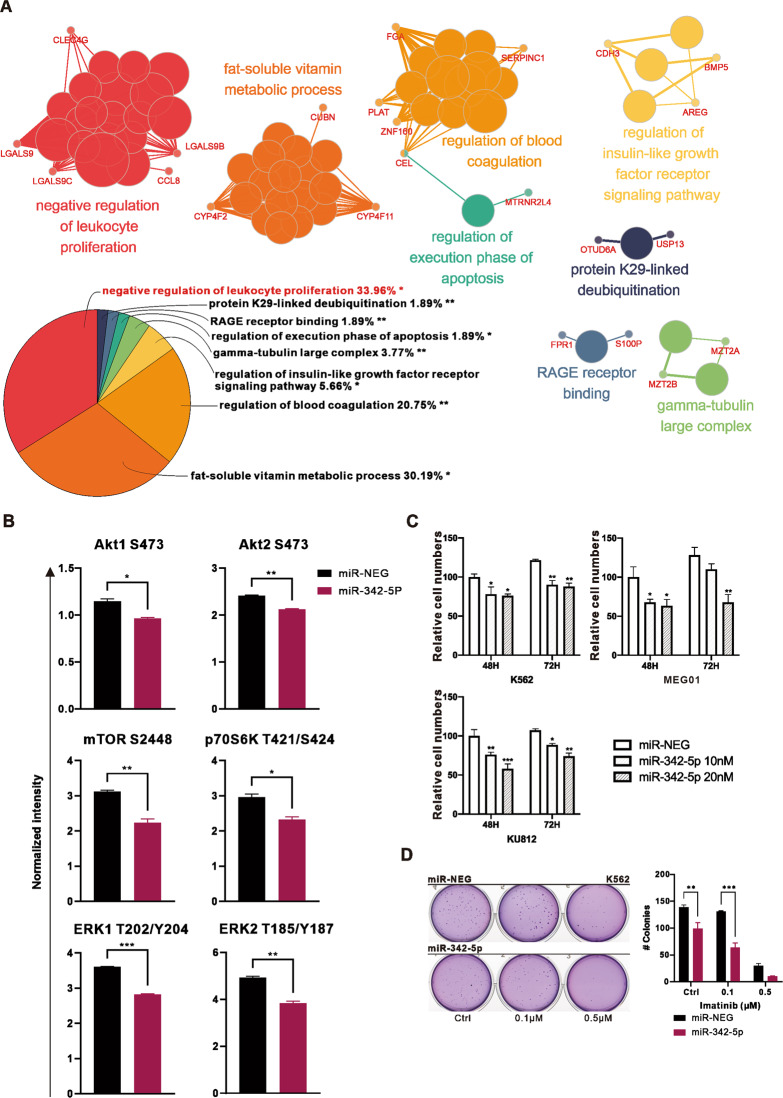
miR-342-5p upregulated-genes are associated with negative regulation of leukocyte proliferation. **A** Presenting miR-342-5p upregulated-gene related ontology using Cytoscape ClueGO plugin. “GO biological processing” was utilized in the analysis. Node clusters represent enriched ontologies or pathways (*p* < 0.05), and the genes associated with the nodes are shown around the cluster. Pie charts present the proportion of each ontology in the collection. **B** The box plots present the normalized intensity of various protein phosphorylation sites with miR-NEG or miR-342-5p expression in the K562 CML cell line using a Human phospho-MAPK array. **C** The effect of miR-NEG and miR-342-5p at 10 or 20 nM on the relative cell numbers at 48 h or 72 h was assessed using Trypan blue assay. **D** Soft agar assay to evaluate colony formation with 0.1 or 0.5 μM imatinib treatment under miR-NEG or miR-342-5p expression (20 nM). The bar chart shows the difference in the number of colony formations. Each data represents mean ± SD from independent experiments performed in triplicate. The student’s *t*-test or One-way ANOVA approach was used to assess the significance. *P < 0.05; **P < 0.01; ***P < 0.001.

Visualized analysis of biological responses related to miR-342-5p upregulated genes showed the highest percentage of “negative regulation of leukocyte proliferation,” followed by “metabolic processes of fat-soluble vitamins” and “regulation of blood coagulation” (Fig. 4A). In addition, “regulation of the insulin-like growth factor receptor signaling pathway” and “regulation of the execution phase of apoptosis” were also associated with the miR-342-5p upregulation-gene signature, suggesting a potential role miR-342-5p in inhibiting cell proliferation and promoting apoptosis.

Analysis of the effects of miR-342-5p on multiple proliferative signaling pathways by Phospho-MAPK arrays showed that phosphorylation of components of the ERK signaling pathway and PI3K-AKT pathway, which are essential for cell proliferation and survival, was significantly reduced (Fig. 4B). In addition, the effects of miR-342-5p on cell viability and colony formation were also analyzed, showing a significant inhibitory effect on the proliferation of K562 (Fig. 4C and D).

### Overexpression of miR-342-5p enhanced imatinib-induced DNA double-strand break and apoptosis in CML

**Fig. 5:**
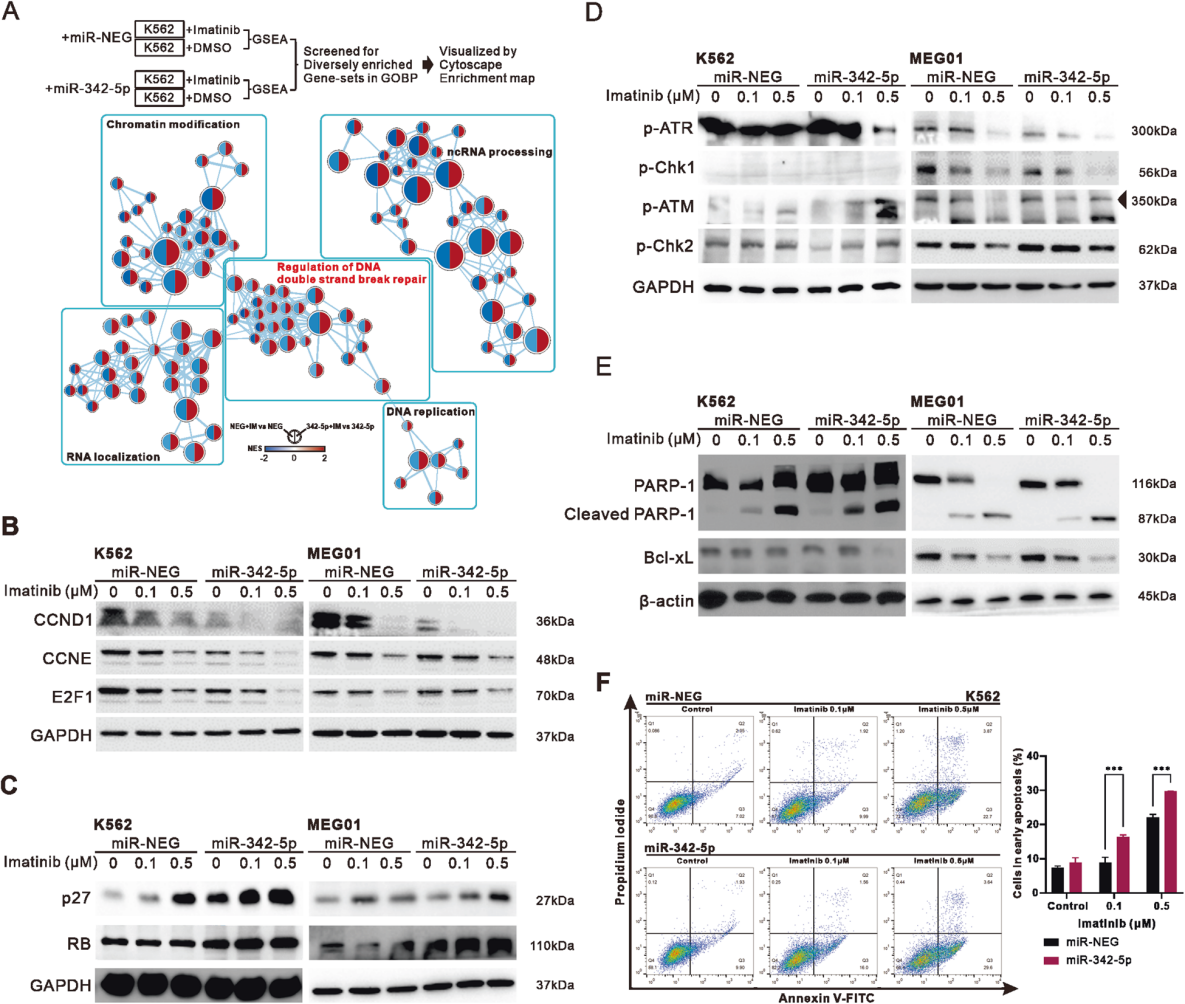
Increased double-stranded DNA breakage response and apoptosis may be a potential mechanism for miR-342-5p inhibition of CML. **A** The enrichment map presented divergent trends of GSEA enrichment with the treatment of 0.5 μM imatinib in miR-NEG or miR-342-5p expression. The width of the edge connected between nodes represents the degree of overlap, and similar nodes are auto-annotated as clusters. GSEA enrichment filtering criteria: *P* < 0.05, FDR < 0.25. **B**–**E** Western blot presents the expression of cell cycle components including CCND1 (B), Cell cycle regulating proteins (**C**), DNA repair components including ATM-chk2 and ATR-chk1 axis (**D**), and apoptosis-associated proteins (**E**) in K562 and MEG01 subjected to imatinib under miR-NEG or miR-342-5p expression (20 nM). **F** Validation of the effect of miR-342-5p on apoptosis in K562 caused by imatinib in the presence of miR-342-5p (20 nM) by Annexin-V/PI method using flow cytometry. The bar chart shows the difference in the percentage of cells at early apoptosis under duplication. The student’s *t*-test or One-way ANOVA approach was used to assess the significance. *P < 0.05; **P < 0.01; ***P < 0.001.

To evaluate the potential differences in response to imatinib treatment in cells with or without miR-342-5p expression, Enrichment maps in Cytoscape were utilized to visualize the comparison of the clustering of nodes with significantly divergent enrichment according to the flow chart [47]. In the group of miR-342-5p overexpression, a significant enrichment of DNA double-strand break repair-related clusters was observed (Fig. 5A). Considering the multiple effects of miR-342-5p on DNA repair ability, cell growth, and apoptosis, we further confirmed the relevant biomarker changes in K562 and MEG01 by western blotting. First, the miR-342-5p significantly inhibited the expression of CCND1, CCNE, and E2F1 (Fig. 5B). Correspondingly, miR-342-5p increased the expression of p27 and Rb (Fig. 5C). Second, evaluation of DNA repair ability using ATR-chk1 and ATM-chk2 showed that imatinib in the presence of miR-342-5p caused the repair mechanism to switch from ATR-chk1 to ATM-chk2 in both K562 and MEG01 (Fig. 5D). Moreover, imatinib treatment under miR-342-5p expression increased cleaved PARP-1 and reduced the anti-apoptotic protein Bcl-xL expression (Fig. 5E), as well as the observation presented in flow cytometry analysis (Fig. 5F), suggesting that miR-342-5p can increase the extent of apoptosis caused by imatinib in CML cells.

### Confirmation of miR-342-5p to enhance the effect of imatinib in vivo

**Fig. 6:**
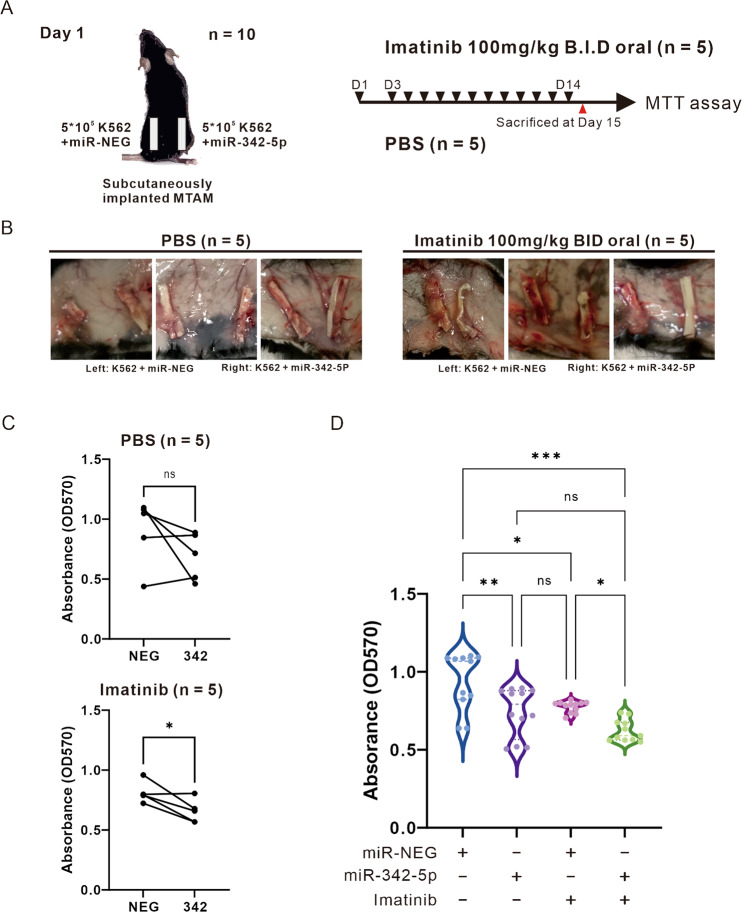
In vivo animal models confirm the ability of miR-342-5p to inhibit CML and enhance the effects of imatinib. **A** Flow diagram of MTAM-system based CML xenograft model. **B** Representative illustration of subcutaneous implantation of MTAM with K562 CML cells in sacrificed mice. **C** Results of MTT assay under a paired analysis of K562 expressing miR-NEG or miR-342-5p. Statistical method: two-tailed Paired *t*-test; ns: not significant difference; *P < 0.05. **D** One-way ANOVA analysis of the results of MTT assay on imatinib treatment with K562 expressing miR-NEG or miR-342-5p; *n* = 6 in each group, ns: not significant difference; *P < 0.05; **P < 0.01; ***P < 0.001. Data were in the form of mean ± SD.

We established an animal model based on subcutaneous implantation of the MTAM system to compare the in vivo growth inhibition of miR-NEG or miR-342-5p-expressing K562 cells with imatinib oral administration [12, 13]. Figure 6A describes the details and procedures of the subcutaneous implantation MTAM model. The maintenance expression of miR-342-5p was confirmed experimentally beforehand (data not shown), and the animals were sacrificed on day 15 to obtain the implanted MTAM. Interestingly, significant neovascularization around MTAM was found in the side receiving imatinib with miR-NEG, while no observation was made in the miR-342-5p side in the same mice (Fig. 6B). Paired comparison of CML cell viability in MTAM from the same animal (left: miR-NEG, right: miR-342-5p) showed lower viability of K562 with miR-342-5p expression and a statistically significant difference in the group receiving imatinib (Fig. 6C). Furthermore, in the combined comparison, the viability of CML cells expressing miR-342-5p was significantly reduced compared to miR-NEG (Fig. 6D, lane 1 vs. 2). This effect was not significant compared to the group with imatinib treatment alone (lane 2 vs. 3), suggesting that miR-342-5p expression already exerted an inhibitory effect on K562. Expression of miR-342-5p significantly increased the inhibitory effect of imatinib on K562 compared to the group receiving concomitant imatinib (lane 3 vs. 4). In addition, the group treated with miR-342-5p and imatinib together significantly inhibited the growth of K562 compared to miR-NEG (lane 1 vs. 4). These results suggest that loss of miR-342-5p affect the ability of imatinib to inhibit CML proliferation in vivo.

### MiR-342-5p upregulated-gene signature in clinical samples reflects prognosis in CML patients

**Fig. 7:**
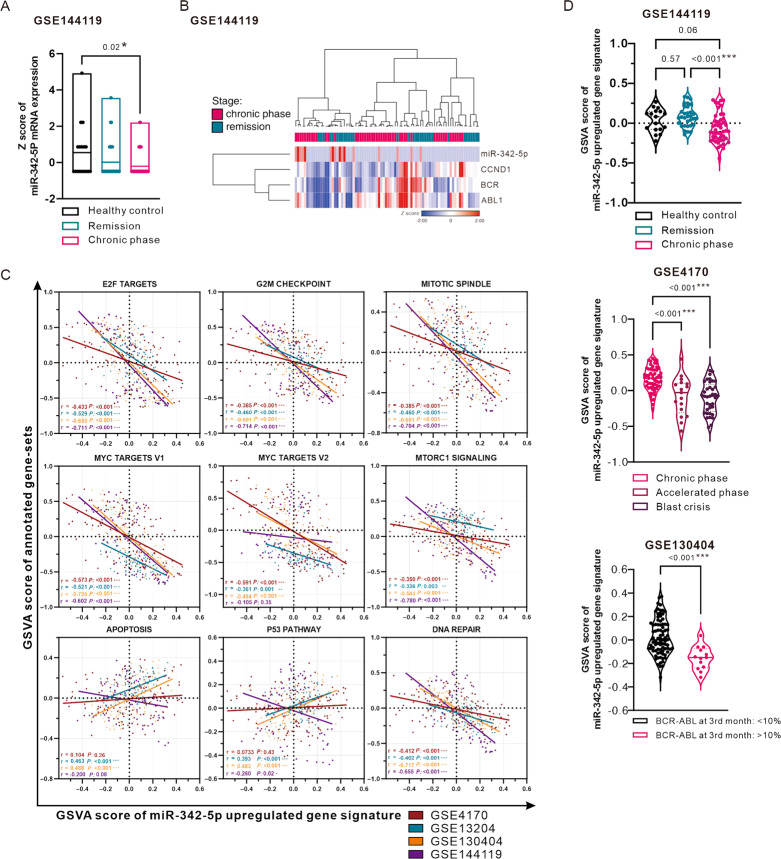
Assessment of miR-342-5p upregulated-gene signature GSVA scoring in clinical CML databases correlates with cell proliferation, apoptosis and DNA repair-related pathways. **A** Box plot illustrating the distribution of normalized miR-342-5p (miR-4664) expression in patients’ blood with different stages in GSE144119. **B** Heat map demonstrating expression of miR-342-5p, CCND1, BCR, and ABL1 in patients’ blood with chronic phase or remission stage in GSE144119. **C** Correlation plot presenting the relationship between GSVA score of miR-342-5p upregulated-gene signature and Hallmark gene-sets associated with cell proliferation, apoptosis, and DNA repair. The correlation was evaluated using Pearson correlation in GSE4170, GSE13204, GSE130404, and GSE144119. ns: non-significant; *P < 0.05; **P < 0.01; ***P < 0.001. **D** Evaluation of miR-342-5P upregulated-gene signature GSVA scores in patients with different stages of disease (GSE144119, upper panel; GSE4170, middle panel) or different BCR-ABL expression (GSE130404, lower panel) in clinical CML patients.

To confirm the distribution of miR-342-5p expression relative to CCND1 and BCR-ABL1 in clinical samples, and to further confirm the correlation between miR-342-5p and cell proliferation, apoptosis and DNA repair gene-set, we used GSE4170, GSE13204, GSE130404, and GSE144119, which were downloaded from the NCBI GEO database. The clinical samples of GSE130404 and GSE144119 were obtained from the PBMC of the patients’ blood samples, which were the same as our clinical samples. GSE4170 was obtained from CD34^+^ cells, and GSE13204 was obtained from the bone marrow of CML patients. Firstly, we confirmed the association between miR-342-5p expression and CML progression in GSE144119, showing that miR-342-5p expression was significantly lower in PBMC from CML patients in the chronic phase (Fig. 7A). Secondly, we confirmed the association of miR-342-5p expression with CCND1, BCR and ABL1 expression. Heat map showed that the distribution of miR-342-5p was opposite to the other three, indicating that the negative association of miR-342-5p with CCND1 in clinical samples (Fig. 7B).

Further evaluation of the GSVA score of miR-342-5p upregulated gene signature (GSVA score of miR-342-5p) in relation to the Hallmark gene-sets showed significant negative correlations with proliferation and DNA repair in the four databases, and mostly positive correlations with apoptosis (Fig. 7C). Similarly, the GSVA score of miR-342-5p was negatively correlated with the expression of BCR mRNA in most databases. We also observed a decrease in BCR mRNA due to miR-342-5p in cellular experiments, suggesting that miR-342-5p may have a role in inhibiting the transcriptional activity of the BCR promoter (supplementary figure). In terms of CML clinical staging and GSVA score of miR-342-5p, patients in the chronic phase were significantly lower than those in healthy control or remission (Fig. 7D, upper panel). GSE4170 provided three stages of CML progression, showing that patients with more aggressive progression had significantly lower GSVA scores of miR-342-5p in CD34^+^ cells (Fig. 7D, middle panel). GSE130404 provided a BCR-ABL expression examination of CML patients at the third month after treatment, showing a significant decrease in GSVA score of miR-342-5p in patients examined as BCR-ABL^+^ (Fig. 7D, lower panel).

**Fig. 8:**
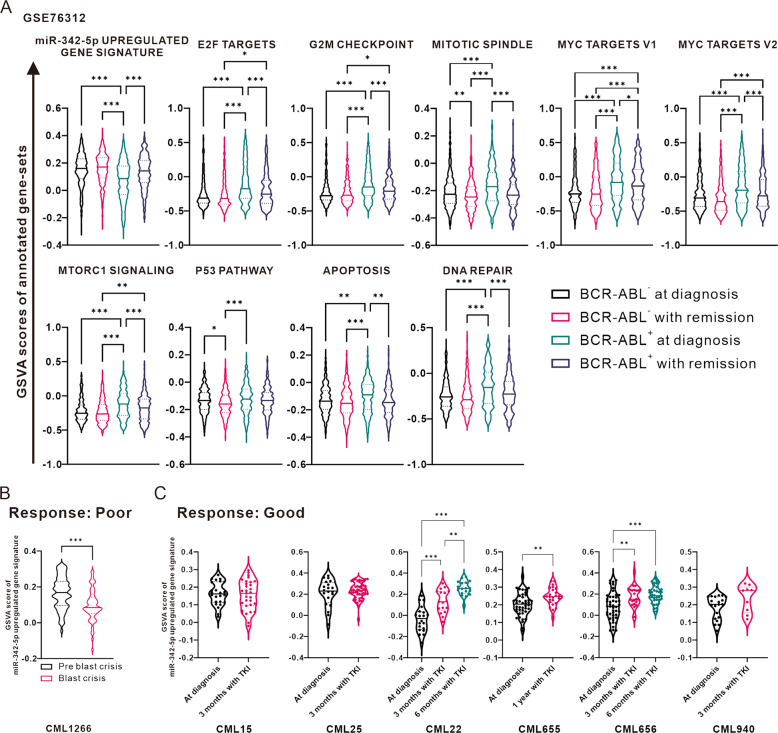
Investigation of GSVA scores of miR-342-5p upregulated-gene signature of single cells from same CML patients with or without remission in GSE76312. **A** The Violin plot shows the distribution of GSVA scores of miR-342-5p upregulated-gene signature, cell proliferation-related biological responses, DNA repair, and apoptosis in the single CML cells with or without BCR-ABL expression at diagnostic or remission states in GSE76312. **B**, **C** GSVA scores of miR-342-5p upregulated-gene signature of single cells from same CML patients with or without remission (Good or Poor). Data were in the form of mean ± SD. One-way ANOVA or Mann-Whitney test were used to assess the significance of statistical differences, respectively. *P < 0.05, **P < 0.01, ***P < 0.001.

Furthermore, we used the CML single-cell RNA sequencing database of GSE76312 to perform pre- and post-treatment differential analysis [25]. The results showed no difference in GSVA scores for miR-342-5p upregulation-gene signature in CML cells without BCR-ABL. Interestingly, there was a significant decrease in the diagnostic group with BCR-ABL^+^ and a rebound in the remission group with BCR-ABL^+^, indicating that miR-342-5p upregulated-gene expression was negatively associated with disease status in patients with BCR-ABL^+^ (Fig. 8A). In addition, proliferation-related biological responses and DNA repair were significantly increased in the BCR-ABL^+^ diagnostic group, which was similar to the results of the bulk transcriptomic analysis. Finally, single-cell sequencing analysis of several CML patients showed that the miR-342-5p upregulation-gene signature of the GSVA score was associated with disease progression. In patients “CML1266” who were not in remission, GSVA scores at the time point of the blast crisis were significantly lower compared to the pre-blast crisis (Fig. 8B), echoing the results of Fig. 7D. Six CML patients were in remission, and four of them had increased GSVA scores after treatment (Fig. 8C), suggesting that detection of miR-342-5p expression may be beneficial in predicting the TKI effect.

## Discussion

Previous studies have shown that miR-342-5p has similar antitumor effects in breast cancer [48], and its downregulation is associated with tamoxifen resistance in breast cancer cells [49, 50]. Our data show for the first time that increasing miR-342-5p intrinsically reduces imatinib resistance in CMLMiR-342-5p acts as a tumor suppressor in CML by suppressing the expression of BCR-ABL and CCND1 (Figs. 2 and 3). BCR-ABL is a crucial factor contributing to the development of CML[51], and CCND1 overexpression in CML is an essential contributor to cell cycle progression [52, 53]. Our results indicate that miR-342-5p can directly interfere with the 3’UTR of CCND1 mRNA to cause a decrease in CCND1 protein expression and may indirectly affect BCR-ABL transcription to reduce its expression. This result suggests that the clinically detected reduction of miR-342-5p may be one of the factors contributing to the aberrant amplification in CCND1 and BCR-ABL expression and further resistance to TKI therapy.

Bioinformatic analysis revealed that miR-342-5p upregulated gene signature was associated with negative regulation of leukocyte proliferation, which could be evidenced by reduced phosphorylation of multiple signaling pathways and inhibition of colony formation (Fig. 4B–D). Similarly, the negative association of GSVA score of miR-342-5p upregulated gene signature with BCR mRNA expression observed in clinical databases implies that miR-342-5p may indirectly affect BCR promoter transcription (supplementary figure), possibly by reducing myc transcriptional activity, which in turn causes reduced BCR-ABL expression (Fig. 7C) [54–56]. In addition, the LGAS9 family encodes a protein, galectin-9, that is thought to cause apoptosis and overcome drug resistance in CML, which may also be one of the mechanisms of drug resistance in CML patients who lose miR-342-5p [57].

Increased double-strand breaks are the leading cause of cell cycle arrest and apoptosis, and different DNA damage response pathways may determine susceptibility to CML [58]. GSEA visualization analysis showed a significant increase in the enrichment of double-stranded DNA breakage regulation in the presence of miR-342-5p expression (Fig. 5A), suggesting that miR-342-5p may affect the DNA repair ability of CML during imatinib treatment, further leading to a switch from the single-stranded repair-induced ATR-chk1 axis to the double-stranded breakage-associated ATM-chk2 axis [59–61]. Morii et al. showed that imatinib inhibits DNA damage checkpoint arrest recovery by inducing sustained activation of ATM/ATR signaling [62]. Skorta et al. reported that imatinib selectively abolished ATM activation induced by drug treatment in BCR-ABL^+^ CML cells [63]. These observations imply that ectopic amplification of BCR-ABL specifically causes selective inactivation of ATM by imatinib through an unknown mechanism. Furthermore, CCND1 expression in the absence of p53 contributes to promoting ATR-Chk1-induced DNA repair, further protecting BCR-ABL^+^ cells from death due to DNA double-strand breaks [64–68]. Given that both K562 and MEG01 are TP53-deficient CML cell lines, inhibition of CCND1 expression by miR-342-5p did lead to ATR-chk1 inhibition with ATM-chk2 activation and subsequent cell apoptosis (Fig. 5D-F) [69, 70]. We suggest that miR-342-5p expression directly or indirectly inhibits CCND1 and BCR-ABL expression, thereby possibly increasing ATM activation by dispersing BCR-ABL-associated ATM repression and reducing CCND1-ATR-Chk1-induced protection, as a result of ATM/ATR switching.

In vivo experiments demonstrated the effect of miR-342-5p in increasing the sensitivity of imatinib to CML and observed the inhibition of angiogenesis (Fig. 6). Considering the association of BCR-ABL and CCND1 expression with the promotion of angiogenesis, miR-342-5p may indirectly affect the ability of the CML periphery to undergo angiogenesis through the inhibition of these two targets [68–70]. Finally, analysis of the CML clinical database revealed that the miR-342-5P upregulated gene signature was negatively correlated with various proliferation-related and DNA repair gene-sets and positively associated with p53 and apoptosis (Fig. 7C). Furthermore, both bulk transcriptome analysis and single-cell RNA sequencing showed that miR-342-5p upregulated gene signature was inversely correlated with the severity of CML and was significantly higher in molecularly responsive patients (Figs. 7D and 8). In conclusion, our findings suggest that measuring miR-342-5p expression has the potential to assess clinical CML disease prognosis and imatinib resistance.

## Supplementary information

Supplemental table 1

Supplemental figure

Supplemental table 2
