# Supplementary material for: Aberrantly reduced expression of miR-342-5p contributes to CCND1-associated chronic myeloid leukemia progression and imatinib resistance
Source: Cell Death Dis. 2021 Oct 5;12(10):908. doi: 10.1038/s41419-021-04209-2 (PMC8492784; doi:10.1038/s41419-021-04209-2)
Supplement: Supplementary file 1 — Supplemental table 1 [file 41419_2021_4209_MOESM1_ESM.pdf]

Characteristics		Patient Number (n = 20)
Age (years)		51.65 (20-88)
Gender		
	Male	12
	Female	8
Splenomegaly (cm)		15.11 (8-21.6)
PB Blast (%)		2.04 (0-11)
BM Blast (%)		3.34 (0.5-5.03)
Eosinophil (%)		3.42 (0-18.4)
Basophil (%)		3.04 (0-13)
WBC count		
	≦ 100,000	7
	> 100,000	13
Platelet count		
	≦ 450,000	13
	> 450,000	7
