# Supplementary material for: Aberrantly reduced expression of miR-342-5p contributes to CCND1-associated chronic myeloid leukemia progression and imatinib resistance
Source: Cell Death Dis. 2021 Oct 5;12(10):908. doi: 10.1038/s41419-021-04209-2 (PMC8492784; doi:10.1038/s41419-021-04209-2)
Supplement: Supplementary file 2 — Supplemental figure [file 41419_2021_4209_MOESM2_ESM.pdf]

A

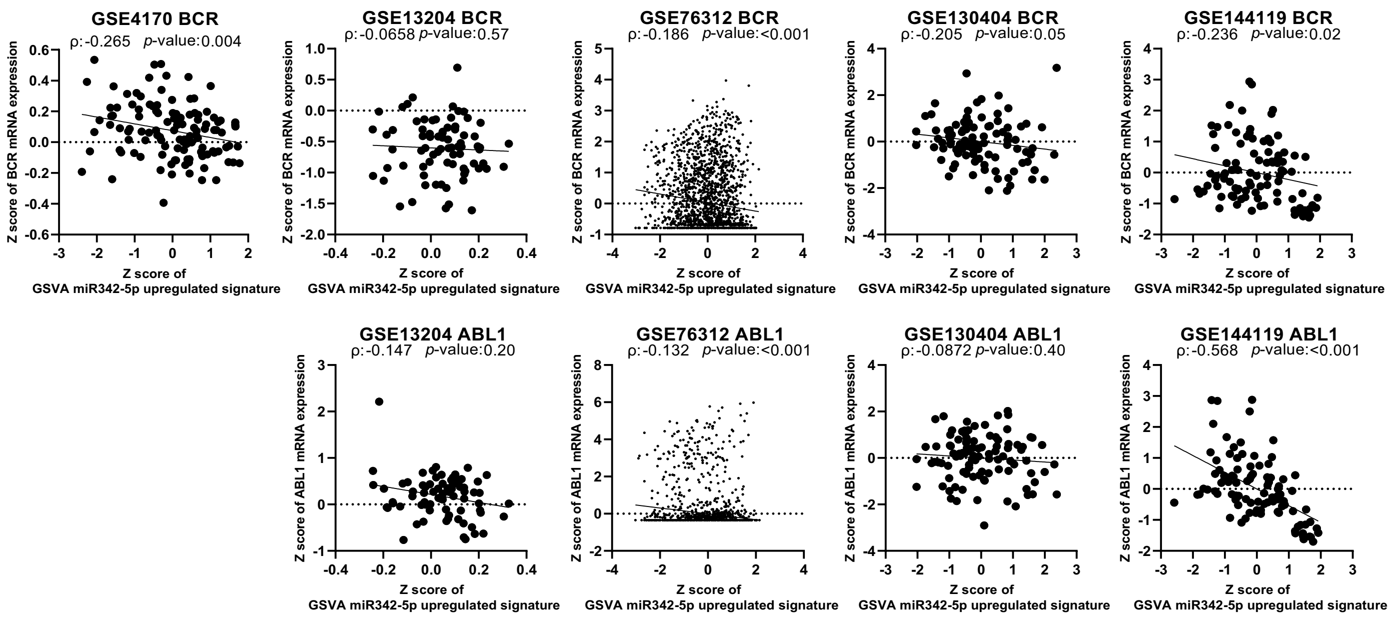

B

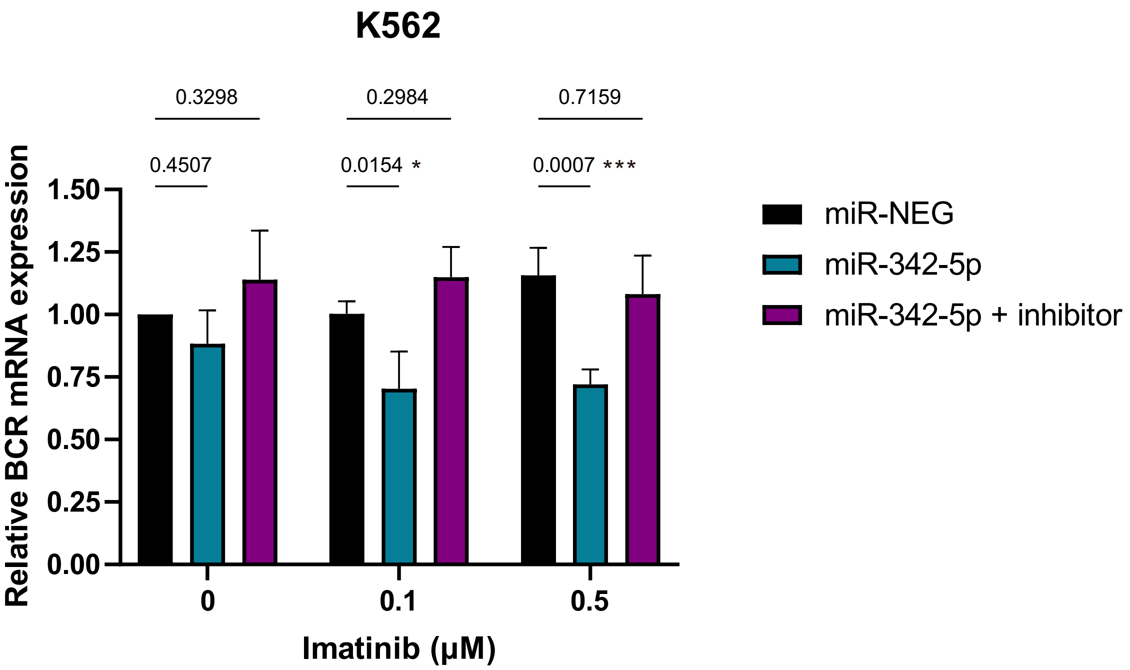

Supppementary Figure Evaluating the relationship between miR-342-5p overexpression and BCR mRNA mRNA expression. (A) Correlation between GSVA score of miR-342-5p upregulated gene signature and normalized mRNA expresssion of BCR and ABL1 in clinical databases. The correlation was evaluated using Spearman correlation. (B) Bar chart presenting the impact of miR-342-5p overexpression on BCR mRNA level analyzed by qPCR. One-way ANOVA was used to assess the significance of statistical differences.
